# Insights into microbiome-triterpenoid correlation in *Poria cocos* via comparative analysis of sclerotial and soil microenvironments

**DOI:** 10.3389/fmicb.2025.1674216

**Published:** 2025-09-17

**Authors:** Mingzhu Zheng, Can Zhong, Gen Pan, Jing Xie, Shuihan Zhang, Jian Jin

**Affiliations:** ^1^Institute of Chinese Medicine Resources, Hunan Academy of Chinese Medicine, Changsha, China; ^2^Hunan Academy of Chinese Medicine, Hunan University of Chinese Medicine, Changsha, China; ^3^Management Committee, Hunan Xiangxi High-Tech Industrial Development Zone, Jishou, China

**Keywords:** *Poria cocos*, high-throughput sequencing, pachymic acid, microbiome, bacteria and fungi

## Abstract

**Introduction:**

*Poria cocos* (*P. cocos*) is a medicinal fungus renowned for its bioactive triterpenoids, particularly pachymic acid. However, the relationship between its specialized microbiota and the accumulation of this key metabolite remains poorly understood.

**Methods:**

This study systematically compared the microbial communities and pachymic acid distribution patterns between *P. cocos* and different soil microenvironments using integrated 16S rRNA/ITS1 sequencing and HPLC-QTOF-MS/MS analysis.

**Results:**

The results revealed significantly lower microbial diversity in *P. cocos* compared to the surrounding soil, with a dominance of *Proteobacteria* and *Ascomycota*, along with specific enrichments of *Burkholderia-Caballeronia-Paraburkholderia* and *Scytalidium*. Pachymic acid was found to accumulate predominantly within the sclerotia, with trace amounts detectable in adjacent soils. Significant positive correlations were identified between pachymic acid and these enriched microbial taxa.

**Discussion:**

These findings indicate that *P. cocos* forms a specialized microenvironment characterized by selective microbial enrichment associated with pachymic acid accumulation, offering valuable insights for optimizing cultivation strategies to improve its medicinal quality.

## 1 Introduction

*Poria cocos* (*P. cocos*), a medicinal and edible macrofungus with over 2000 years of documented therapeutic use, is renowned for its diuretic, sedative, and immunomodulatory properties (Zhang et al., 2019; He et al., 2025; Guo et al., 2025). Pachymic acid is one of its principal bioactive components and has attracted considerable attention due to its remarkable antitumor (Yang et al., 2022; Jiang and Fan, 2020), anti-inflammatory, and immunoregulatory effects (Li et al., 2015). Recent advances in omics technologies have enabled preliminary elucidation of the biosynthetic pathways of triterpenoids in *P. cocos* through genomic and metabolomic studies (Liu et al., 2024; Noushahi et al., 2024; Liu et al., 2023). Nevertheless, the microbial regulatory mechanisms involved in sclerotium formation, particularly the relationship between pachymic acid biosynthesis and microbial community interactions, remain poorly understood.

In studies of biological-environmental interactions, both plants and macrofungi exhibit complex interactions with microbial communities. Research on plants has demonstrated that terpenoid compounds can resist pest infestation and enhance plant resistance (Pétriacq et al., 2017a; Huang et al., 2019; Zhong et al., 2022), while also functioning as signaling molecules to regulate rhizosphere microbial communities (Sugiyama et al., 2016; Bouwmeester et al., 2025). Distinct from plant root systems, most fungi establish unique microecosystems through their mycelial networks, facilitating material exchange and signal transmission with the surrounding substrate (Xu et al., 2023). In edible mushroom cultivation systems, *Pleurotus ostreatus* cultivation relies on *Actinobacteria* and *Firmicutes* to degrade lignocellulose, providing carbon sources for mycelial growth (Suarez et al., 2020). *Stropharia rugosoannulata* can enrich beneficial fungi such as *Mortierella* and *Cladosporium*, which not only suppress pathogens but also enhance soil nutrient levels through secondary metabolite production (Tang et al., 2022). Additionally, studies on *Morchella* have revealed a close correlation between the enrichment of carbon-fixing *Chloroflexi* and yield improvement, potentially through providing carbon skeleton precursors for host secondary metabolism (Tan et al., 2021). Regarding medicinal fungi, the synthesis of triterpenoids in *Ganoderma lucidum* shows significant correlation with rhizosphere microorganisms (Ren et al., 2020a; Chang et al., 2018). These findings provide important references for understanding the microbial regulation mechanisms in medicinal fungi.

Unlike typical mushrooms or polypores, the medicinal part of *P. cocos* is not the fruiting body but rather the sclerotium formed by tightly aggregated mycelia. This unique structure likely relies on distinct microbial interaction patterns. While numerous studies have investigated microorganism-secondary metabolite relationships in medicinal plants such as Astragalus (Lin et al., 2022; Zhiyong et al., 2024; Li et al., 2021), Salvia (Zhang et al., 2023) and Ginkgo (Jia et al., 2025), Astragalus, similar research on medicinal fungi - particularly regarding *P. cocos* - remains completely unexplored.

By integrating high-throughput sequencing with high-performance liquid chromatography-quadrupole/time-of-flight mass spectrometry (HPLC-QTOF-MS/MS), this study systematically characterizes the structural features of the *P. cocos* sclerotium microecosystem and its correlation with pachymic acid synthesis. The findings provide novel theoretical insights into the correlation mechanisms between medicinal fungi and associated microbiota, while offering scientific support for ecological cultivation techniques aimed at enhancing *P. cocos* quality through microecological regulation.

## 2 Materials and methods

### 2.1 Cultivation of *Poria cocos*

The present study was conducted at the Chinese Medicine Resources Experimental Base of the Hunan Academy of Chinese Medicine, Changsha, Hunan Province, China (N 28°12′09.10″, E 112°45′03.05″). *P. cocos* spawn was provided by the Jingzhou *P. cocos* Association of Hunan, China, and cultivation was carried out using pine wood segments (60 cm in length) as the substrate. Four experimental groups were established: spawn & woods, woods-only, spawn-only, and bare soil as the control, with six replicates per group. For cultivation, trenches (50 cm deep × 90 cm wide) were dug, and wood segments were horizontally placed at 2-meter intervals. In the spawn + woods group, the spawn bags were cross-cut with three incisions each vertically and horizontally before being placed between two tightly adjacent pine wood segments and secured with polyethylene film. The woods-only and spawn-only groups contained only wood segments or spawn bags, respectively, while the bare soil group remained untreated. All trenches were backfilled with topsoil.

### 2.2 Harvesting of samples

**Figure 1:**
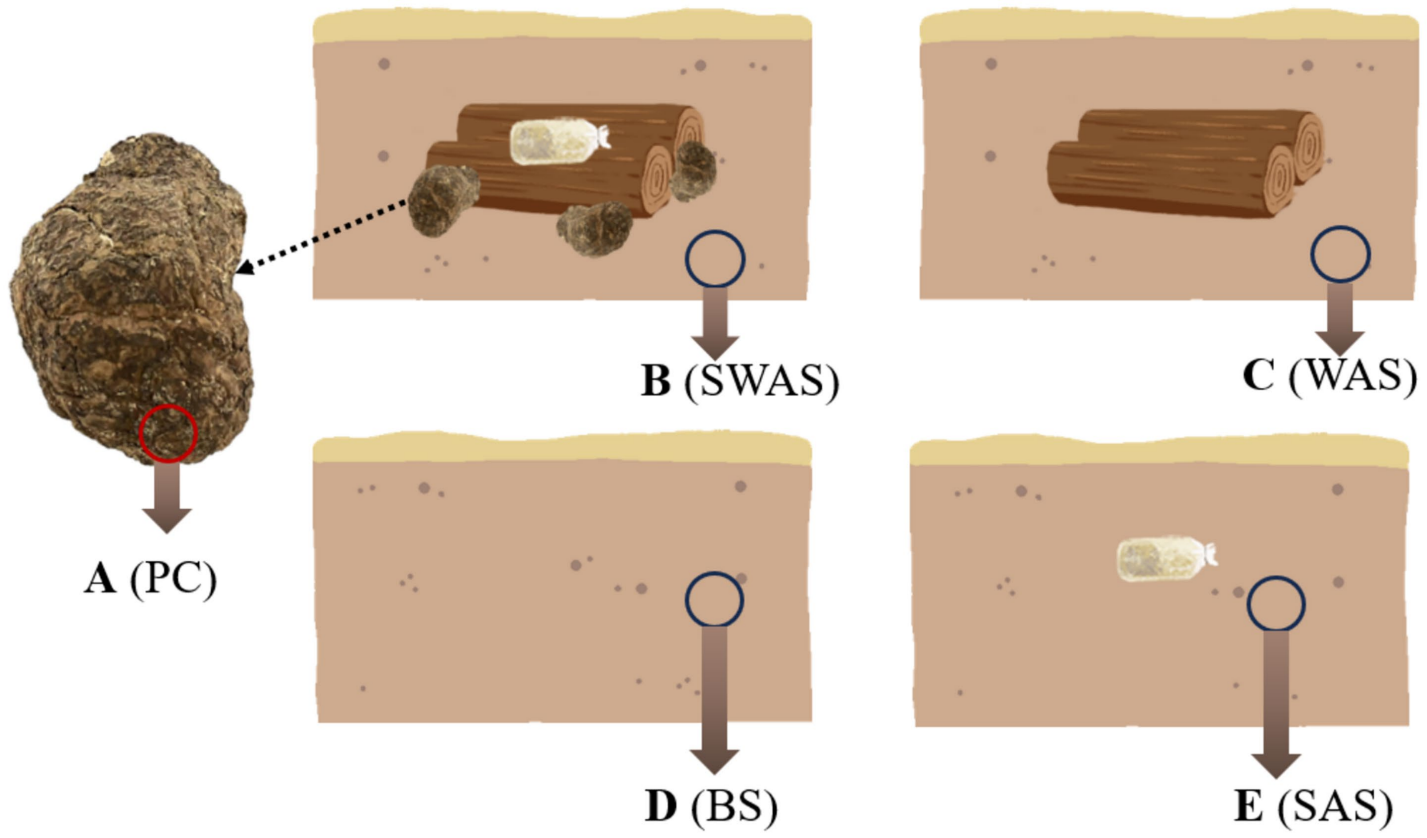
Schematic diagram of sampling locations in *Poria cocos* and the surrounding soil. **(A)** (PC), *Poria cocos*; **(B)** (SWAS), Spawn’ and Wood Adiacent Soil; **(C)** (WAS), Wood Adiacent Soil; **(D)** (BS), Bare Soil; **(E)** (SAS), Spawn Adiacent Soil.

After 9 months of cultivation, sampling was conducted by carefully excavating the topsoil to collect mature *P. cocos* specimens. For the *P. cocos* samples, the sclerotia were carefully unearthed, rinsed with distilled water to remove surface soil, and then wiped with 75% ethanol. The outer peel was aseptically excised using a sterilized scalpel as the representative sample. Soil samples were collected from each treatment group at a distance of 10 cm from either the *P. cocos*, spawn bags, or wood segments, as illustrated in Figure 1. Following collection, all soil and fungal samples were systematically divided into two aliquots: the first was immediately flash-frozen in liquid nitrogen and stored at −80°C for subsequent microbial community analysis through ITS and 16S rRNA gene sequencing, while the second aliquot was preserved for precise quantification of pachymic acid content.

### 2.3 DNA extraction, PCR amplification and sequencing

According to the manufacturer’s instructions, DNA extraction was performed using a DNA extraction kit. For bacterial 16S rRNA amplification, the primers 341F (CCTAYGGGRBGCASCAG) and 806R (GGACTACNNGGGTATCTAAT) (Ferreira et al., 2024) were used. For fungal ITS1 region amplification, the primers ITS1F (CTTGGTCATTTAGAGGAAGTAA) and ITS2R (GCTGCGTTCTTCATCGATGC) (Bukavina et al., 2022) were employed. After purification, quantification, and normalization of the PCR products, 16S rRNA/ITS amplicon sequencing was performed on an Illumina NovaSeq 6,000 platform by Wuhan Frasergen Bioinformatics Co., Ltd. (Wuhan, China). The raw image data files were then converted into raw sequencing reads through base calling. The results were saved in FASTQ format, which includes sequence data and corresponding quality scores. All sequences were deposited at the Sequence Read Archive (SRA) of the National Center for Biotechnology Information (NCBI) under project accession number PRJNA1310015.

### 2.4 Bioinformatics analysis

The QIIME 2 software platform was used to process the data obtained from Illumina HiSeq amplicon sequencing. Briefly, the reads were processed and applied to DADA2 pipeline for the assignment of amplicon sequence variants (ASVs). Before application to DADA2, the fungal sequences were trimmed by the plugin of ITSxpress. The DADA2 workflow includes filtering, dereplication, chimera identification, and merging of the paired-end sequences. Taxonomic assignment of the ASVs was performed using the SILVA 128 database for bacteria and the UNITE database for fungi. The RDP Classifier algorithm was employed for sequence alignment analysis. To assess sequencing depth sufficiency and sampling completeness, Good’s coverage estimator was computed. Alpha diversity metrics (including observed ASVs, Shannon index, and Faith’s phylogenetic diversity) and beta diversity analyses (using UniFrac distances) were subsequently calculated to evaluate within-sample and between-sample microbial diversity patterns, respectively. Beta diversity was visualized by principal coordinate analysis (PCoA). In order to further explore the differences in microbial community structure among different groups, partial least squares discrimination analysis (PLS-DA) was applied.

### 2.5 Extraction and HPLC-QTOF-MS/MS analysis of pachymic acid

The collected samples were placed in a constant-temperature drying oven and continuously dried at 55°C until reaching constant weight, then ground into uniformly-sized powder. Precisely 1.00 ± 0.01 g of the powdered sample was weighed and mixed with 5 mL of methanol for ultrasound-assisted extraction. The extraction process was conducted in an ultrasonic bath with 220 W power and 50 kHz frequency for 15 min. After allowing the extract to stand for 20 min, the supernatant was filtered through a 0.22 μm organic phase microporous membrane. Chromatographic analysis was performed using HPLC-QTOF-MS/MS. Acetonitrile and pure water were used as mobile phases A and B, respectively. The injection volume was 10 μL with a solvent flow rate of 1 mL/min, using an InterSustain C18 column (5 μm, 4.6 × 250 mm, GL Sciences Inc., Tokyo, Japan) maintained at 20°C. The solvent gradient program was set as follows:0–10 min: 51% A; 10–15 min: 51–86% A; 15–25 min: 86–100% A; 25–35 min: 100% A; 35–40 min: 100–51% A; 40–50 min: 51% A. Mass spectrometry conditions employed negative ion mode with a scan range of m/z 100–1,500.

### 2.6 Statistical analysis

The quantitative determination of pachymic acid was done in six repetitions and expressed as mean ± standard deviation. Differences among groups were analyzed statistically using one-way analysis of variance. Statistical analyses of different samples were performed using the Origin statistical software. Pearson’s correlation coefficient was utilized to analyze the correlation. *p* < 0.05 was considered to indicate a statistically significant difference.

## 3 Results

### 3.1 Microbial community composition

**Figure 2:**
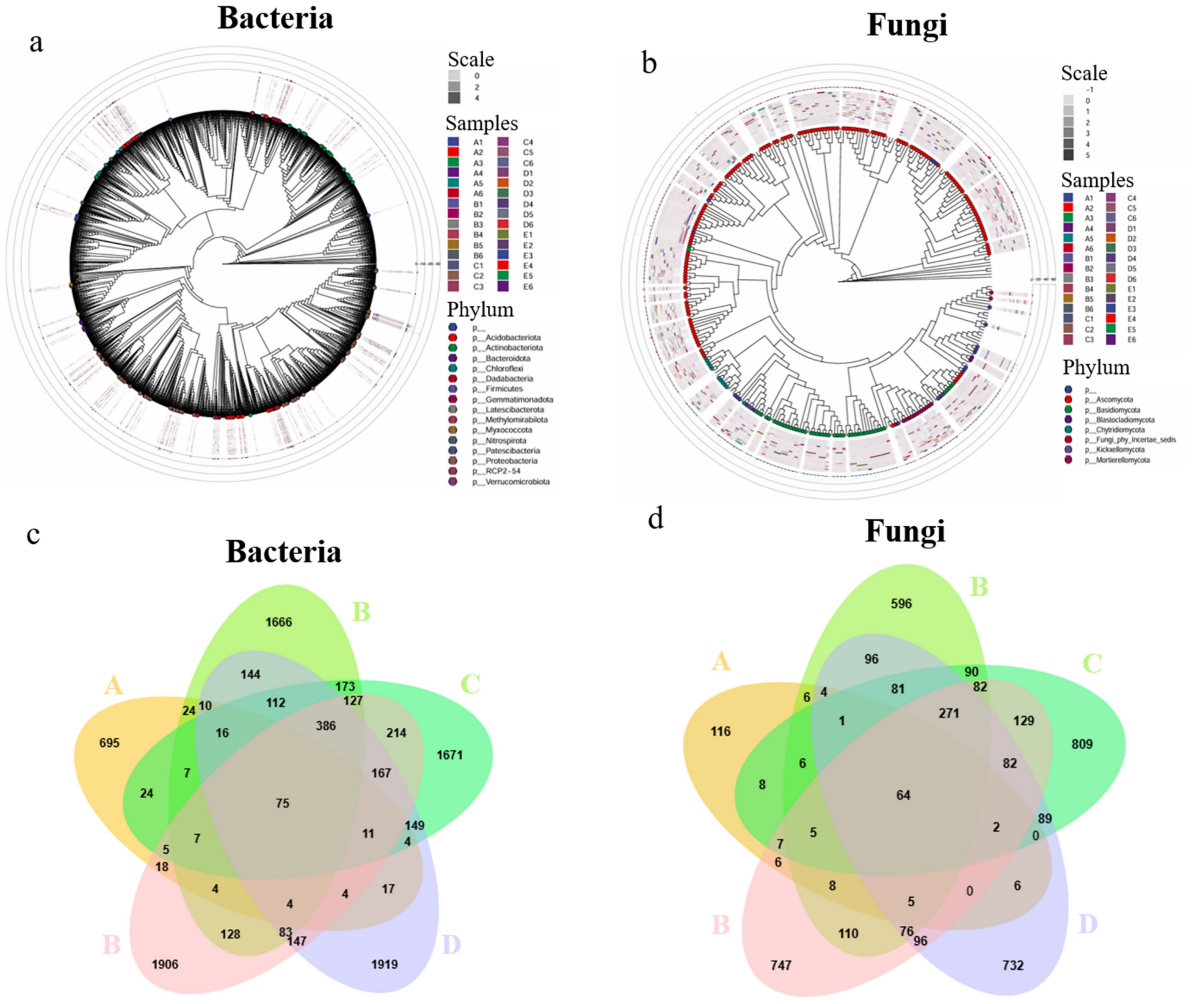
Phylogenetic trees of fungal **(a)** and bacterial **(b)** genera at the genus level. Venn diagrams showing shared and unique fungal **(c)** and bacterial **(d)** ASVs among samples. A, *Poria cocos*; B, spawn and wood adjacent soil; C, wood adjacent soil; D, bare soil; E, spawn adjacent soil.

This study conducted a systematic taxonomic analysis of microbial communities across five sample groups. A total of 39 bacterial phyla and 820 genera were identified, along with 16 fungal phyla and 587 genera. Significant differences were observed in the relative abundance of various taxonomic units among sample groups, with their phylogenetic relationships illustrated in Figure 2a (bacterial genus level) and Figure 2b (fungal genus level).

High-throughput sequencing yielded 17,578 high-quality ASVs. Among these, bacterial ASVs accounted for 13,175 (75.0%), while fungal ASVs comprised 4,403 (25.0%). Microbial community analysis revealed that the core bacterial and fungal ASVs shared across all samples numbered only 75 and 64, respectively (Figures 2c,d), indicating significant compositional differences among microenvironments.

Microbial composition analysis across sample groups revealed that *P. cocos* (A) contained 925 bacterial ASVs and 244 fungal ASVs, while the spawn & wood adjacent soil (B) harbored 2,966 bacterial ASVs and 1,501 fungal ASVs. The wood-adjacent soil (C) showed 3,148 bacterial ASVs and 1,726 fungal ASVs, with bare soil (D) containing 3,248 bacterial ASVs and 1,605 fungal ASVs. The spawn adjacent soil (E) demonstrated the highest microbial diversity with 3,286 bacterial ASVs and 1,690 fungal ASVs (Figures 2c,d). These results demonstrate that *P. cocos* harbors a distinct microbial community composition compared to surrounding soil environments, with significantly lower microbial diversity.

### 3.2 Beta diversity analysis of microbial community

**Figure 3:**
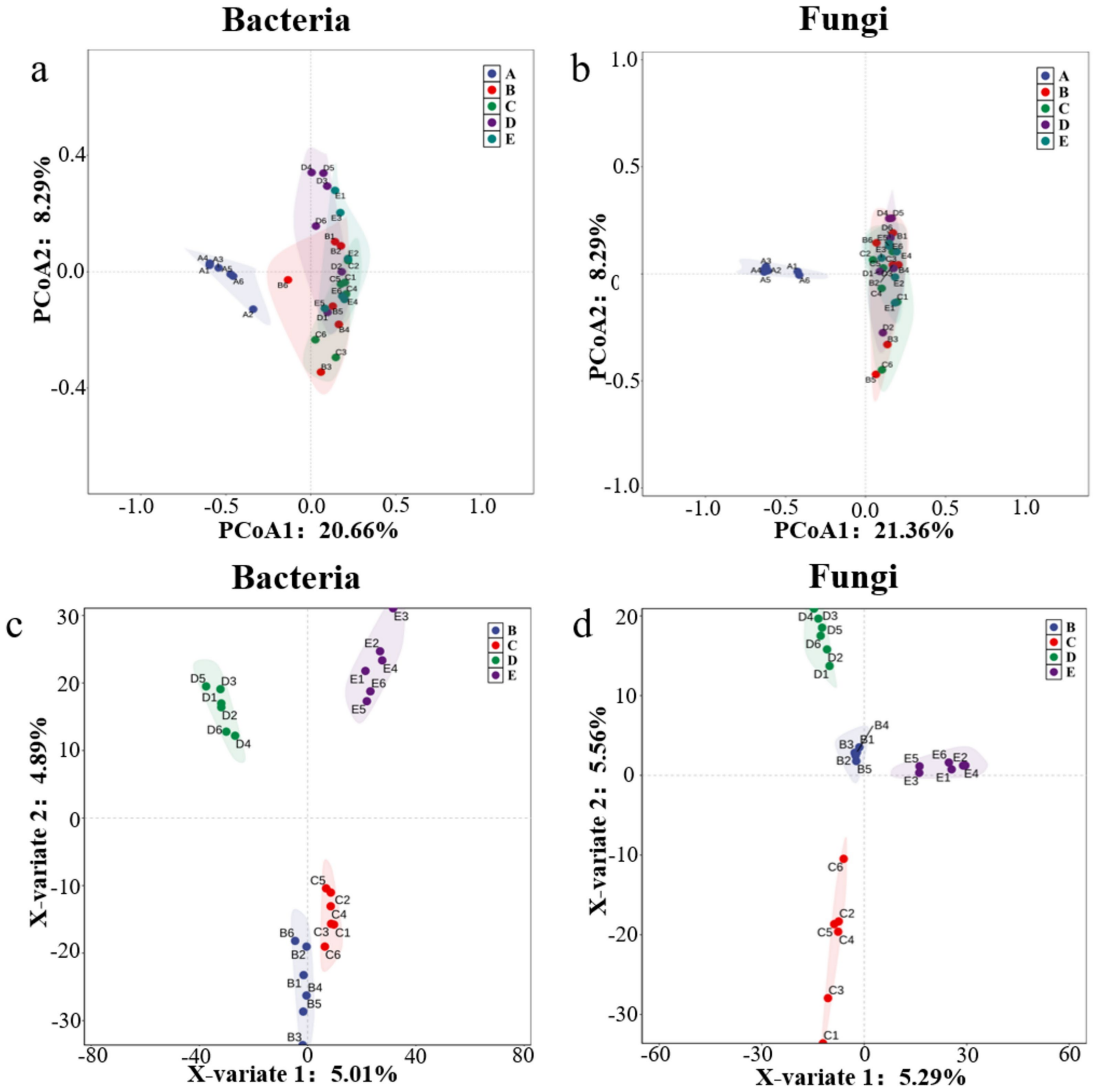
Multivariate analysis of microbial beta diversity. **(a)** Principal coordinates analysis (PCoA) of bacterial communities; **(b)** PCoA of fungal communities; **(c)** partial least squares-discriminant analysis (PLS-DA) of bacterial communities; **(d)** PLS-DA of fungal communities. A, *Poria cocos*; B, spawn and wood adjacent soil; C, wood adjacent soil; D, bare soil; E, spawn adjacent soil.

Multivariate statistical analysis revealed significant differences in microbial communities among sample groups. Principal coordinate analysis (PCoA) based on Bray-Curtis distance showed that the first two axes (PCoA1 and PCoA2) of bacterial communities cumulatively explained 31.79% of the total variance (PCoA1: 20.66%; PCoA2: 8.61%), while those of fungal communities explained 29.65% (PCoA1: 21.36%; PCoA2: 8.29%). The analysis demonstrated that *P. cocos* samples (A) were separated from soil sample groups (B-E) in the coordinate space, while different soil groups showed partial overlap but still exhibited clustering tendencies (Figures 3a,b). To validate this pattern, partial least squares-discriminant analysis (PLS-DA) was performed to examine inter-group differences among soil samples. The results showed significant separation of all groups under the supervised model (Figures 3c,d). The consistency between these two analytical approaches indicates that *P. cocos* possesses a distinct microbial community structure, while differences among soil groups exist but with relatively blurred boundaries.

### 3.3 Alpha diversity analysis of microbial community

**Figure 4:**
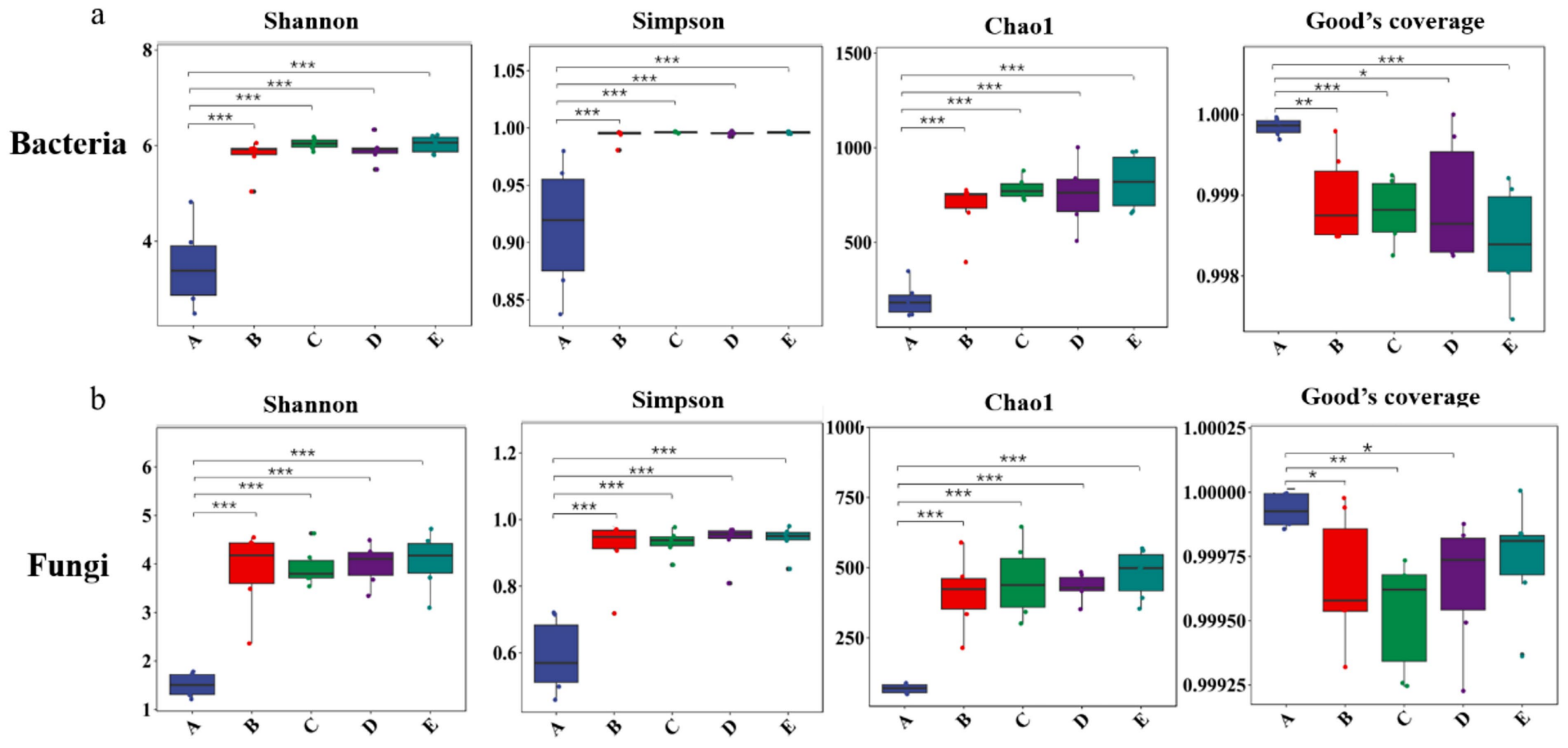
Alpha diversity of microbial communities. **(a)** bacteria; **(b)** fungi. A, *Poria cocos*; B, spawn and wood adjacent soil; C, wood adjacent soil; D, bare soil; E, spawn adjacent soil. Statistical significance was determined by Student’s *t*-test. *p<0.05; **p<0.01; ***p<0.001.

The alpha diversity analysis reflects microbial community richness and diversity within samples. Shannon, Simpson, and Chao1 indices collectively revealed that all soil groups (B-E) exhibited substantially higher microbial richness and diversity compared to *P. cocos* samples (A) (*p* < 0.001), while no significant differences were observed among the various soil groups themselves. The high Goods coverage values (>99.9%) across all samples confirmed adequate sequencing depth, ensuring comprehensive detection of predominant microbial taxa and reliable representation of both bacterial and fungal community compositions. Notably, the boxplot visualization (Figure 4) clearly illustrated these patterns, with *P. cocos* (A) consistently showing the lowest diversity values for both bacteria (Figure 4a) and fungi (Figure 4b), while the four soil microenvironment groups (B-E) formed a cluster of higher diversity values without statistically significant inter-group variations. These results further corroborate the distinct nature of the *P. cocos* microbiome relative to its surrounding soil environments.

### 3.4 Bacterial community composition analysis

**Figure 5:**
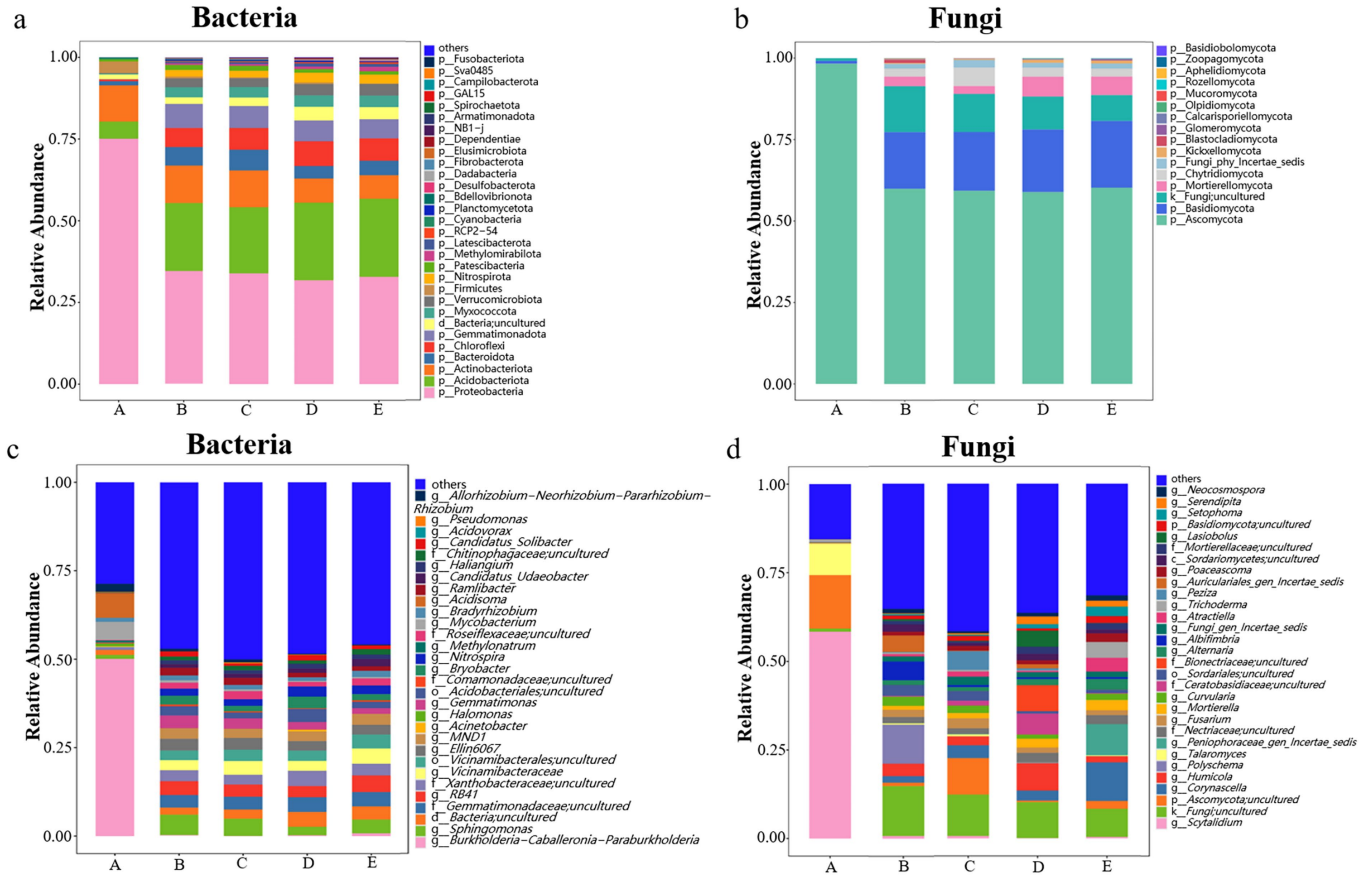
Bacteria abundance in different samples: **(a)** at phylum level, top 30 phylum shown, rest as others; **(c)** at genus level, top 30 genus shown, rest as others; Fungi abundance in different samples: **(b)** at phylum level; **(d)** at genus level, top 30 genus shown, rest as others. A, *Poria cocos*; B, spawn & wood adjacent soil; C, wood adjacent soil; D, bare soil; E, spawn adjacent soil.

The bacterial community analysis revealed that *Proteobacteria*, *Actinobacteriota*, and *Acidobacteriota* were the dominant phyla (relative abundance >5% in all groups) (Figure 5a; Supplementary Table S1). Notably, *Proteobacteria* was the most abundant phylum across all samples, with its relative abundance in *P. cocos* (A) being more than double that in soil groups (approximately 75% vs. 30–35%). The *P. cocos* group exhibited distinct bacterial community characteristics, showing significantly higher relative abundances of *Proteobacteria* and *Firmicutes*, but lower abundances of *Actinobacteriota*, *Bacteriodota*, *Chloroflexi*, and *Gemmatimonadota* compared to soil groups. Interestingly, soil samples containing pine wood segments (B and C) demonstrated higher relative abundances of *Proteobacteria*, *Actinobacteriota*, and *Gemmatimonadota*, but lower abundances of *Acidobacteriota* and *Chloroflexi* compared to wood-free soil samples (D and E).

At the genus level (Figure 5b; Supplementary Table S2), *P. cocos* (A) displayed a unique profile dominated by *Burkholderia-Caballeronia-Paraburkholderia* (50.02%), *Acidisoma* (6.93%), and *Mycobacterium* (5.10%). In contrast, the four soil groups shared more similar community structures with greater overall richness. For instance, Group B was characterized by distinct dominant genera including *Sphingomonas* (5.82%), *RB41*, *Gemmatimonas* (3.59%), and several uncultured taxa from *Gemmatimonadaceae*, *Xanthobacteraceae*, and *Vicinamibacteraceae* families. The higher relative abundance of *Sphingomonas* and *Gemmatimonas* in Group B compared to other soil groups suggests potential microenvironment-specific bacterial adaptations.

### 3.5 Fungal community composition analysis

The fungal community characteristics (Figure 5c) mirrored those of the bacterial communities: *P. cocos* samples (A) exhibited distinct characteristics at the phylum level, with *Ascomycota* dominating (98.29%) while *Basidiomycota* and *Mortierellomycota* were present in minimal abundance (<1%). This contrasted sharply with soil groups (B-E), where *Ascomycota* accounted for 58–60% of the community (Supplementary Table S3). Beyond the dominant phyla, soil samples harbored various low-abundance phyla including *Mortierellomycota*, *Chytridiomycota*, and Fungi_phy_Incertae_sedis. Notably, bare soil (D) and spawn-only adjacent soil (E) showed significantly higher relative abundance of *Basidiomycota* (19–20%), potentially reflecting substrate-specific influences.

At the genus level (Figure 5d; Supplementary Table S4), Group A was characterized by dominant genera *Scytalidium* (58.37%), uncultured *Ascomycota* (15.11%), and *Talaromyces* (8.86%). In contrast, soil groups demonstrated clear substrate-dependent community patterns: Group B featured *Polyschema* as its characteristic genus, Group C was dominated by *Peziza*, Group D showed enrichment of *Humicola*, while Group E was represented by *Corynascella*. These differences further substantiate the significant impact of growth substrates on fungal community structure.

### 3.6 Pachymic acid distribution and chemical profiling

**Figure 6:**
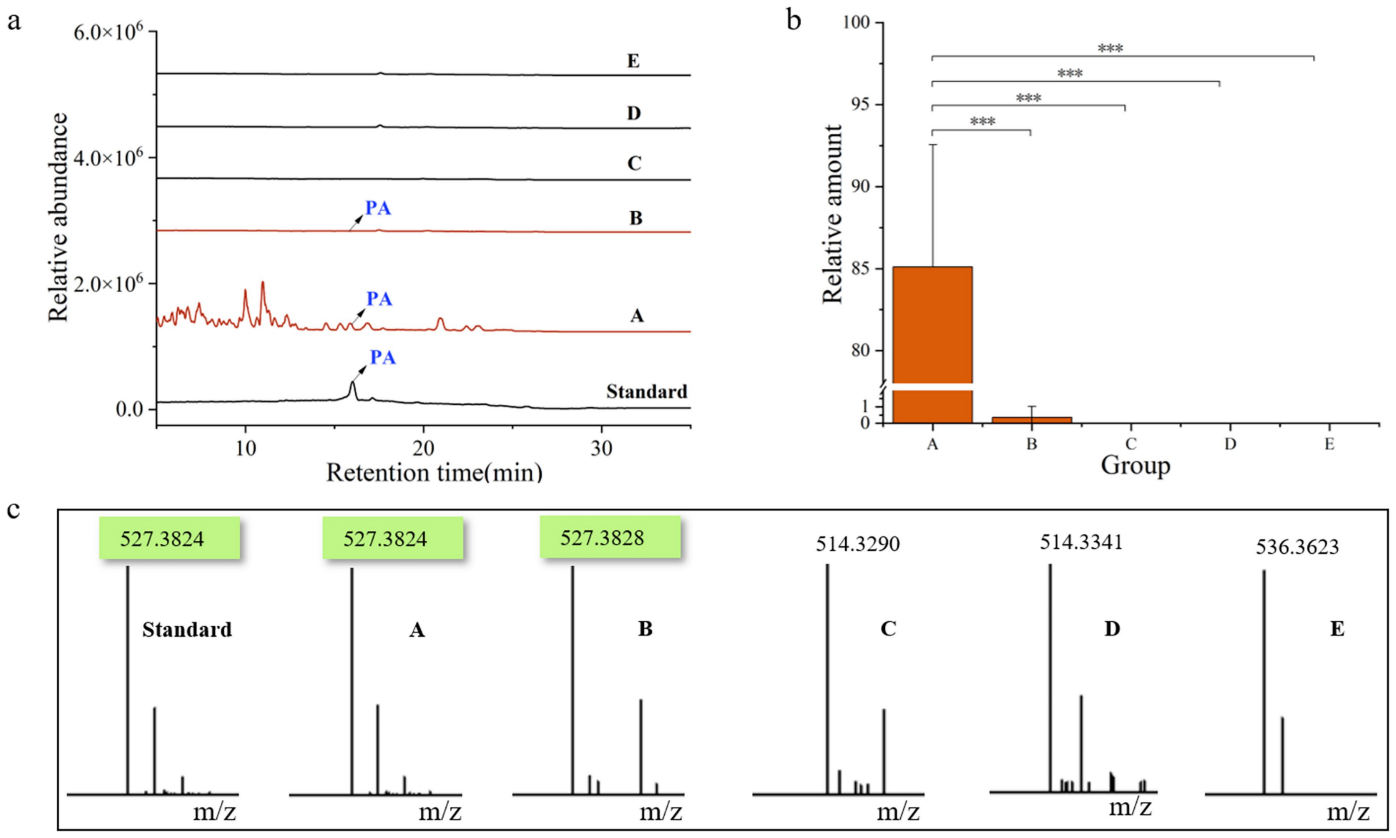
Chemical profiling of pachymic acid in different sample groups. **(a)** Total ion chromatograms of the six samples. **(b)** Relative amount of pachymic acid in each group of samples. **(c)** Corresponding mass spectrometry profiles of the six samples. Standard, pachymic acid; A, *Poria cocos*; B, spawn & wood adjacent soil; C, wood adjacent soil; D, bare soil; E, spawn adjacent soil. *p<0.05; **p<0.01; ***p<0.001.

After 9 months of cultivation, quantitative analysis of pachymic acid content across all samples (Figure 6) revealed distinct accumulation patterns. *P. cocos* samples (Group A) exhibited substantial pachymic acid accumulation, while only trace amounts were detected in the spawn & wood adjacent soil (B). No pachymic acid was detected in other soil groups (C-E). These findings demonstrate that pachymic acid is a specific metabolite of *P. cocos*, primarily synthesized through its endogenous metabolic activity rather than by environmental microorganisms. The trace detection in Group B likely originates from either active secretion or passive diffusion from *P. cocos*.

### 3.7 Correlation analysis between Pachymic acid content and microbial communities

**Figure 7:**
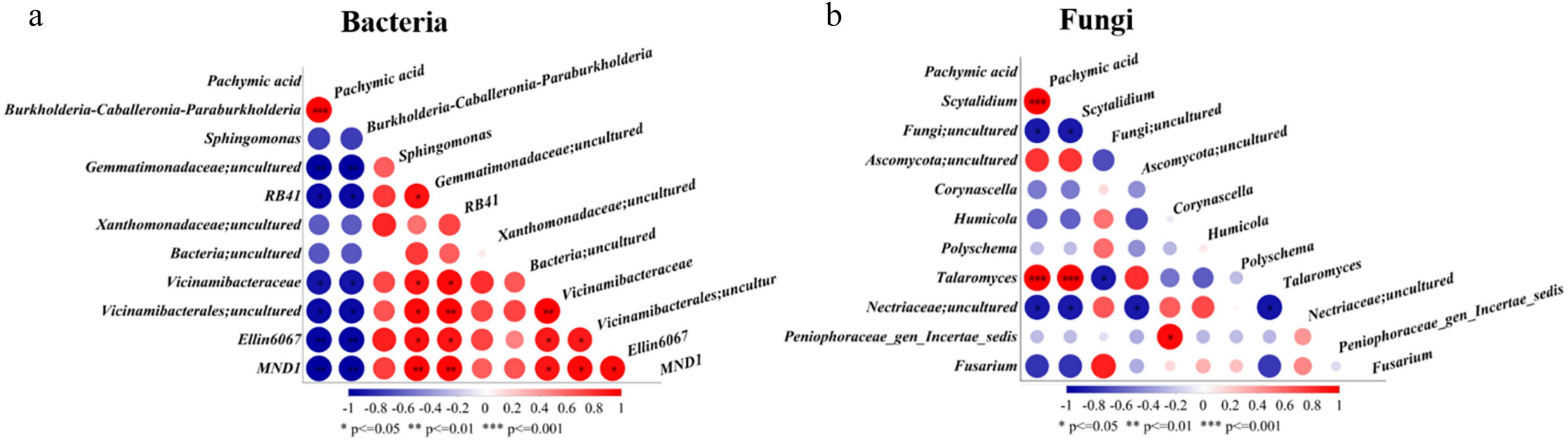
Correlations between pachymic acid content and top 10 microbial genera: **(a)** bacterial, **(b)** fungal. Red indicates positive correlations while blue shows negative correlations, with color intensity representing correlation strength. Circle size corresponds to statistical significance: *p < 0.05; **p < 0.01; ***p < 0.001.

To investigate the potential associations between pachymic acid accumulation and microbial communities, we performed correlation analysis between pachymic acid content and the top 10 most abundant bacterial and fungal genera across samples (Figure 7). The analysis revealed significant positive correlations between pachymic acid content and *Burkholderia-Caballeronia-Paraburkholderia* (*p* < 0.001) among bacterial communities, while showing significant negative correlations with *Gemmatimonadaceae*, RB41, *Vicinamibacteraceae*, *Vicinamibacterales*, Ellin6067 and MND1 (*p* < 0.05). Notably, these negatively correlated bacterial genera exhibited strong positive inter-genus relationships. For fungal communities, pachymic acid content demonstrated significant positive correlations with both *Scytalidium* and *Talaromyces* (*p* < 0.01), which themselves showed an exceptionally strong positive correlation (*p* < 0.001). These results suggest that pachymic acid accumulation is closely associated with selective enrichment of specific microbial taxa in the sclerotium microenvironment, suggesting a potential association between host metabolite production and microbial community composition.

## 4 Discussion

This study reveals the unique microbial composition of *P. cocos* and its interaction mechanisms with the environment. Alpha and beta diversity analyses demonstrated significantly lower microbial diversity (Shannon, Simpson and Chao1 index, *p* < 0.001) in *P. cocos* samples (Group A) compared to each soil group. PCoA analysis (31.79% variance explained for bacteria, 29.65% for fungi) showed distinct clustering of *P. cocos* communities, a pattern strongly correlated with the specific distribution of pachymic acid. This suggests that host secondary metabolites may play a crucial role in microbial community selection. Similar mechanisms have been demonstrated in various plant-microbe interaction systems. For instance, *Arabidopsis thaliana* shapes specific rhizosphere microbiomes through secreted triterpenoids (Huang et al., 2019; Pétriacq et al., 2017b), while tomatoes recruit actinobacteria to suppress pathogens by secreting riboflavin and 3-hydroxyflavones (Yang et al., 2023).

Analysis of microbial community composition revealed that the dominant bacterial phyla across all samples included *Proteobacteria*, *Actinobacteria*, and *Acidobacteria*, consistent with their widespread distribution in soil ecosystems (Maestre et al., 2015). Notably, *P. cocos* exhibited significant enrichment of *Proteobacteria* and *Firmicutes*, while *Actinobacteriota*, *Bacteroidota*, and *Chloroflexi* were more abundant in soil groups. This differential distribution pattern implies host-mediated active selection, potentially through suppression of actinobacterial resource competition or antibiotic effects to optimize its microecological environment (Janssen, 2006; Yun et al., 1997). At the genus level, *Burkholderia-Caballeronia-Paraburkholderia* showed prominent enrichment and significant correlation with pachymic acid content. These lignin-degrading microbes can produce secondary metabolic precursors such as ferulic acid, vanillic acid, and malic acid, which provide carbon sources for *P. cocos* growth (Smith et al., 2007; Akita et al., 2016; Morya et al., 2021). *Paraburkholderia*, a monophyletic clade derived from *Burkholderia*, has been shown to promote plant growth and enhance disease resistance (Cao et al., 2025; Mitter et al., 2017; Ledger et al., 2016). Similar to its role in inducing the accumulation of flavonoids and hydroxycinnamic acid in broccoli (Jeon et al., 2021), the strain may further affect the synthesis of medicinal active ingredients by regulating the secondary metabolism of *P. cocos*. Additionally, acid-tolerant *Acidisoma* may thrive in *P. cocos* ‘s acidic microenvironment rich in triterpenoids (Yu et al., 2023). The selective enrichment of functional microbes like nitrogen-fixing *Sphingomonas* collectively constitutes the unique microbiome signature of *P. cocos* (Li et al., 2023; Li et al., 2025; Kämpfer et al., 2025). Particularly noteworthy is a recent discovery that *Streptomyces* in citrus rhizospheres harbors abundant terpenoid synthesis gene clusters, potentially promoting host monoterpene accumulation by gene encoding providing 1-deoxy-D-xylulose-5-phosphate synthase pathway intermediates (Su et al., 2023), offering new theoretical insights into the interaction between pachymic acid synthesis and microbial communities.

Wood-containing groups (B and C) showed increased *Proteobacteria*, *Actinobacteriota*, and *Gemmatimonadota* abundance with concurrent reduction of *Acidobacteriota* and *Chloroflexi*, potentially reflecting carbon partitioning from lignocellulose degradation (Mendes et al., 2021; Gavande et al., 2021; Koubová et al., 2023). The phosphorus-solubilizing *Gemmatimonas* (Kang et al., 2015; Zhu et al., 2024) may gain a competitive advantage in the high-carbon, low-phosphorus wood substrate environment, consistent with observations in *Ganoderma* cultivation systems (Ren et al., 2020b). However, these correlations require validation through metabolomic approaches to elucidate direct carbon utilization mechanisms.

The fungal communities were dominated by *Ascomycota* across all samples, with significantly higher relative abundance in *P. cocos* compared to soil groups. In contrast, *Basidiomycota*, *Mortierellomycota*, and *Chytridiomycota* were more prevalent in soils. This distinct distribution pattern may stem from antibiotic secretion by *P. cocos* suppressing competitors, analogous to the antifungal strategies employed by *Tricholoma matsutake* (Oh et al., 2016; Liu et al., 2021). The dominant fungal genera in *P. cocos*, *Scytalidium* and *Talaromyces*, showed significant correlations with pachymic acid content. *Scytalidium* is a thermophilic fungal genus. Co-culture of *Scytalidium* parasiticum and *Ganoderma* boninense induces the production of antimicrobial metabolites (e.g., alkaloids and flavonoids) active against Ganoderma (Ahmad et al., 2020), which may help suppress *Ganoderma*-induced basal stem rot (Goh et al., 2016). *Talaromyces* has been reported to possess abundant secondary metabolite gene clusters including terpene synthases (Zhai et al., 2016; Weber et al., 2015), while some *Ascomycetes,* like *Bulgaria inquinans,* can synthesize triterpenoid acids, such as betulinic and ursolic acids. While some Ascomycetes (e.g., *Bulgaria inquinans*) have been shown to produce triterpenoid acids (Zhang et al., 2005). This suggests potential direct involvement of *P. cocos*-associated fungi in triterpenoid acid biosynthesis. Soil groups exhibited substrate-dependent variations at the genus level, with distinct dominant taxa (*Polyschema* in B, *Peziza* in C, *Humicola* in D, and *Corynascella* in E), reflecting the differential impacts of fungal inoculation, spawn bags and wood segments.

The unique spatial distribution of pachymic acid - abundant in *P. cocos*, only a small amount in the surrounding soil, but it is not detected in the control soil at all. This strongly supports active host selection of specific microbiota, consistent with known roles of terpenoids in microbial community regulation (Pichersky and Raguso, 2018). While this study has revealed significant associations between the microbial community and metabolic products in *P. cocos*, the underlying functional mechanisms require further validation. Future investigations should employ strain isolation and multi-omics approaches to systematically elucidate the *P. cocos*-microbiota-triterpenoid interaction network. These studies will provide more robust theoretical foundations for microbiome-mediated cultivation optimization of medicinal fungi.

## 5 Conclusion

This study revealed that *P. cocos* establishes a unique microecosystem by enriching specific microbial taxa, particularly *Proteobacteria* and *Ascomycota*, with the *Burkholderia-Caballeronia-Paraburkholderia* and *Scytalidium* showing significant correlations with the specific accumulation of pachymic acid. In contrast, surrounding soil groups exhibited higher microbial diversity with distinct compositional differences observed among various treatment groups. The findings suggest that the secretion of triterpenoid compounds in *P. cocos* coincides with the presence of specific microbial taxa, while pine wood substrates further optimize this metabolic microenvironment by promoting lignin-degrading microbial communities. These results advance our understanding of fungus-microbe interactions in medicinal species and provide insights for cultivation optimization.

## Data Availability

The datasets presented in this study can be found in online repositories. The names of the repository/repositories and accession number(s) can be found in the article/Supplementary material.
