# Supplementary material for: Insights into microbiome-triterpenoid correlation in Poria cocos via comparative analysis of sclerotial and soil microenvironments
Source: Front Microbiol. 2025 Sep 17;16:1674216. doi: 10.3389/fmicb.2025.1674216 (PMC12484010; doi:10.3389/fmicb.2025.1674216)
Supplement: Supplementary file 1 [file Table_1.docx]

**Supplementary Materials**

Table S1. Top 10 phylum in terms of relative abundance of bacteria (%).

| **Taxonomy** | **A** | **B** | **C** | **D** | **E** |
| --- | --- | --- | --- | --- | --- |
| Proteobacteria | 75.03 | 34.58 | 33.90 | 31.76 | 32.82 |
| Actinobacteriota | 11.10 | 11.63 | 11.26 | 7.38 | 7.20 |
| Acidobacteriota | 5.27 | 20.79 | 20.22 | 23.77 | 23.89 |
| Bacteroidota | 1.26 | 5.63 | 6.36 | 3.82 | 4.46 |
| Chloroflexi | 0.53 | 5.86 | 6.62 | 7.55 | 6.82 |
| Gemmatimonadota | 0.13 | 7.37 | 6.75 | 6.40 | 5.86 |
| Bacteria;uncultured | 1.42 | 2.03 | 2.60 | 4.18 | 3.71 |
| Myxococcota | 0.26 | 3.12 | 3.23 | 3.49 | 3.51 |
| Verrucomicrobiota | 0.19 | 2.66 | 2.66 | 3.48 | 3.58 |
| Firmicutes | 3.50 | 0.56 | 0.31 | 0.49 | 0.13 |
| Others | 1.31 | 5.78 | 6.09 | 7.67 | 8.01 |

Table S2. Top 30 genus in terms of relative abundance of bacteria (%).

| **Taxonomy** | **A** | **B** | **C** | **D** | **E** |
| --- | --- | --- | --- | --- | --- |
| Burkholderia-Caballeronia-Paraburkholderia | 50.02 | 0.18 | 0.05 | 0.23 | 0.79 |
| Sphingomonadaceae;uncultured | 0.04 | 0.07 | 0.12 | 0.06 | 0.08 |
| Bacteria;uncultured | 1.42 | 2.03 | 2.60 | 4.18 | 3.71 |
| Gemmataceae;uncultured | 0.00 | 0.01 | 0.00 | 0.00 | 0.03 |
| RB41 | 0.01 | 3.81 | 3.45 | 3.16 | 4.69 |
| Xanthomonadaceae;uncultured | 0.00 | 0.06 | 0.10 | 0.01 | 0.08 |
| Vicinamibacteraceae | 0.06 | 2.76 | 3.88 | 2.80 | 4.30 |
| Vicinamibacteraceae;uncultured | 0.00 | 0.01 | 0.09 | 0.07 | 0.06 |
| Ellin6067 | 0.01 | 3.31 | 3.33 | 2.67 | 2.76 |
| MND1 | 0.01 | 2.94 | 2.54 | 2.73 | 3.08 |
| Acinetobacter | 0.00 | 0.00 | 0.00 | 0.00 | 0.00 |
| Halomonas | 0.72 | 0.00 | 0.00 | 0.00 | 0.00 |
| Gemmatimonas | 0.06 | 3.59 | 2.99 | 2.17 | 1.64 |
| Acidobacteriales;uncultured | 0.03 | 2.68 | 1.65 | 3.69 | 1.79 |
| Comamonadaceae;uncultured | 0.17 | 0.49 | 0.33 | 0.21 | 0.44 |
| Bryobacter | 0.15 | 2.60 | 1.54 | 3.30 | 1.73 |
| Nitrospira | 0.03 | 1.97 | 1.86 | 2.84 | 2.41 |
| Methylonatrum | 0.44 | 0.00 | 0.00 | 0.00 | 0.00 |
| Roseiflexaceae;uncultured | 0.19 | 1.68 | 2.29 | 1.29 | 2.01 |
| Mycobacterium | 5.10 | 0.66 | 0.51 | 0.31 | 0.42 |
| Bradyrhizobium | 1.13 | 1.39 | 1.25 | 1.01 | 1.74 |
| Acidisoma | 6.93 | 0 | 0 | 0 | 0 |
| Ramlibacter | 0.07 | 2.13 | 2.01 | 1.28 | 1.27 |
| Candidatus_Solibacter | 0.01 | 1.51 | 0.66 | 1.50 | 1.03 |
| Acidovorax | 0.03 | 0.00 | 0.00 | 0.00 | 0.00 |
| Pseudomonas | 0.19 | 0.02 | 0.34 | 0.18 | 0.01 |
| Allorhizobium-Neorhizobium-Pararhizobium-Rhizobium | 2.29 | 0.72 | 0.82 | 0.26 | 0.29 |
| Others | 30.88 | 65.38 | 67.57 | 66.05 | 65.63 |

Table S3. Top 10 phylum in terms of relative abundance of fungi (%).

| **Taxonomy** | **A** | **B** | **C** | **D** | **E** |
| --- | --- | --- | --- | --- | --- |
| Ascomycota | 98.29 | 59.90 | 59.28 | 58.89 | 60.24 |
| Basidiomycota | 0.85 | 17.40 | 18.06 | 19.18 | 20.49 |
| Fungi;uncultured | 0.83 | 14.07 | 11.67 | 10.14 | 7.92 |
| Mortierellomycota | 0.02 | 2.96 | 2.42 | 6.08 | 5.69 |
| Chytridiomycota | 0.00 | 2.46 | 5.65 | 2.81 | 2.45 |
| Fungi_phy_Incertae_sedis | 0.00 | 1.45 | 2.36 | 1.44 | 1.57 |
| Kickxellomycota | 0.00 | 0.26 | 0.28 | 0.94 | 0.91 |
| Blastocladiomycota | 0.00 | 0.97 | 0.00 | 0.01 | 0.00 |
| Glomeromycota | 0.00 | 0.03 | 0.07 | 0.08 | 0.33 |
| Calcarisporiellomycota | 0.00 | 0.06 | 0.05 | 0.23 | 0.15 |
| Others | 0.00 | 0.43 | 0.16 | 0.21 | 0.25 |

Table S4. Top 30 genus in terms of relative abundance of fungi (%).

| **Taxonomy** | **A** | **B** | **C** | **D** | **E** |
| --- | --- | --- | --- | --- | --- |
| Scytalidium | 58.37 | 0.74 | 0.67 | 0.07 | 0.41 |
| Fungi;uncultured | 0.83 | 14.07 | 11.67 | 10.14 | 7.92 |
| Ascomycota;uncultured | 15.11 | 0.96 | 10.30 | 0.43 | 2.23 |
| Corynascella | 0.00 | 1.84 | 3.62 | 2.85 | 10.88 |
| Humicola | 0.03 | 3.50 | 2.48 | 7.67 | 1.72 |
| Polyschema | 0.00 | 11.01 | 0.03 | 0.14 | 0.05 |
| Talaromyces | 8.86 | 0.38 | 0.55 | 0.05 | 0.23 |
| Peniophoraceae_gen_Incertae_sedis | 0.00 | 0.00 | 0.12 | 0.00 | 8.76 |
| Nectriaceae;uncultured | 0.00 | 1.80 | 1.59 | 2.74 | 2.55 |
| Fusarium | 0.25 | 2.02 | 2.85 | 1.51 | 1.37 |
| Mortierella | 0.01 | 1.11 | 1.46 | 2.45 | 2.89 |
| Curvularia | 0.05 | 2.57 | 2.11 | 1.19 | 1.79 |
| Ceratobasidiaceae;uncultured | 0.00 | 0.22 | 1.38 | 5.91 | 0.19 |
| Sordariales;uncultured | 0.00 | 3.06 | 2.71 | 0.71 | 0.93 |
| Bionectriaceae;uncultured | 0.00 | 0.01 | 0.01 | 7.30 | 0.03 |
| Alternaria | 0.04 | 1.34 | 1.14 | 1.73 | 2.99 |
| Albifimbria | 0.04 | 5.26 | 0.55 | 0.49 | 0.53 |
| Fungi_gen_Incertae_sedis | 0.00 | 1.45 | 2.36 | 1.44 | 1.57 |
| Atractiella | 0.00 | 0.65 | 1.45 | 0.39 | 3.87 |
| Trichoderma | 0.81 | 0.42 | 0.47 | 0.17 | 4.47 |
| Peziza | 0.00 | 0.18 | 5.30 | 0.55 | 0.00 |
| Auriculariales_gen_Incertae_sedis | 0.00 | 4.66 | 0.15 | 1.17 | 0.01 |
| Poaceascoma | 0.00 | 1.05 | 1.32 | 1.10 | 2.42 |
| Sordariomycetes;uncultured | 0.00 | 2.05 | 0.79 | 1.80 | 1.11 |
| Mortierellaceae;uncultured | 0.01 | 1.00 | 0.47 | 2.03 | 1.77 |
| Lasiobolus | 0.00 | 0.47 | 0.16 | 4.52 | 0.05 |
| Basidiomycota;uncultured | 0.01 | 1.06 | 1.41 | 0.62 | 1.94 |
| Setophoma | 0.01 | 0.37 | 0.35 | 1.22 | 2.65 |
| Serendipita | 0.00 | 0.24 | 0.38 | 2.16 | 1.71 |
| Neocosmospora | 0.00 | 1.23 | 0.59 | 1.02 | 1.48 |
| Others | 15.55 | 35.29 | 41.59 | 36.41 | 31.50 |
